# How drought and ploidy level shape gene expression and DNA methylation in *Phragmites australis*

**DOI:** 10.1007/s00299-025-03585-9

**Published:** 2025-08-12

**Authors:** Kristina Kuprina, Kerstin Haldan, Stepan Saenko, Mohamed Safwaan Gulam, Jürgen Kreyling, Martin Schnittler, Manuela Bog

**Affiliations:** 1 Institute of Botany and Landscape Ecology, University of Greifswald, Soldmannstraße 15, 17489 Greifswald, Germany; 2 Institute of Mathematics and Computer Science, University of Greifswald, Walther-Rathenau-Straße 47, 17489 Greifswald, Germany; 3 Institute of Horticultural Production Systems, Leibniz University Hannover, Herrenhäuser Straße 2, 30419 Hannover, Germany

**Keywords:** Common reed, Paludiculture, RNA-seq, Epigenetics, Ploidy, Water stress

## Abstract

**Key message:**

Key drought-response genes regulate saccharopine, mevalonate, water-stress pathways, and cell wall remodeling. Ploidy level influences gene expression under drought and non-stress conditions. Octoploids overall exhibit lower methylation than tetraploids.

**Abstract:**

Drought stress significantly affects plant physiology and growth, yet the molecular mechanisms underlying drought responses remain poorly understood. In this study, we investigate how tetraploid and octoploid *Phragmites australis* (common reed), a key species in wetland ecosystems and paludiculture, respond to drought at the transcriptional and epigenetic levels. Using RNA-seq, we identify changes in gene expression after 20 and 30 days of drought and assess methylation-sensitive amplification polymorphism (MSAP) over 50 days of drought. Transcriptomic analysis reveals that key drought-response genes are shared between ploidy levels, including those involved in the saccharopine pathway, water deprivation response, cell wall remodeling, and the mevalonate pathway. Drought suppresses photosynthetic genes, with *PsbP* downregulated by up to 32-fold. Ploidy level influences gene expression under both drought and non-stress conditions, highlighting distinct adaptive strategies. In control samples, gene expression differed between ploidy levels, with octoploids upregulating genes related to translation and metabolism, while tetraploids activate genes involved in cell wall modification and transmembrane transport. Prolonged drought increases DNA methylation variability, though no significant correlation was detected between methylation levels and drought duration. Methylation differences are more pronounced between ploidy levels, with octoploids exhibiting lower overall methylation. These findings highlight the complex interactions between gene expression, epigenetic modifications, and polyploidy in drought response and provide a theoretical framework for future selection, hybridization, and conservation initiatives.

**Supplementary Information:**

The online version contains supplementary material available at 10.1007/s00299-025-03585-9.

## Introduction

Drought is one of the most significant consequences of climate variability and anthropogenic effects (Haile et al. 2020). It is projected to increase in both frequency and intensity in the coming decades, particularly in mid-latitudes (Murray and Ebi 2012; Taylor et al. 2013; Rodell et al. 2018). These prolonged disruptions in water availability pose severe threats to ecosystems, particularly wetlands, which are highly dependent on stable hydrological conditions.

Among wetland plants, *Phragmites australis* (Cav.) Trin. ex Steud., or common reed, plays a significant role in wetland ecosystems, which is not only essential for maintaining biodiversity and ecosystem functions but also serves as a key species in paludiculture—a sustainable wetland use practice (Timmermann et al. 2006; Becker et al. 2020; Geurts et al. 2020; Lahtinen et al. 2022; Martens et al. 2023). This practice involves cultivating wetland plants such as *P. australis* under waterlogged conditions, helping to conserve carbon stocks, reduce greenhouse gas emissions, and support renewable biomass production, making it an effective tool for mitigating climate change (Wichtmann et al. 2020; Martens et al. 2022).

*P. australis* stands out for its exceptional tolerance to environmental stress. Its adaptability is demonstrated by its broad ecological range, thriving in both freshwater and saline habitats. The species shows optimal salinity tolerance between 0 and 16 parts per thousand (ppt), with certain lineages capable of enduring salinity levels up to 56 ppt (Lissner et al. 1999; Gao et al. 2012; Achenbach et al. 2013). In addition, *P. australis* exhibits remarkable flexibility in water levels, with optimal growth occurring within a water table range of − 30 cm to + 70 cm and survival in water depths from − 6 m to + 2.3 m (Haslam 1972; Engloner 2009; Cui et al. 2010). Despite this adaptability, even brief periods of water deficiency can lead to reduced plant growth (Pagter et al. 2005). Rewetted peatlands, including those used in paludiculture, experience greater water table fluctuations (Kreyling et al. 2021), which in combination with climate change leads to drought conditions in naturally moist habitats.

The high adaptability and phenotypic plasticity of *P. australis* is closely linked to its high genetic variation—traits commonly associated with widely distributed species (Clevering et al. 2001; Meyerson et al. 2016; Eller et al. 2017). Different genotypes of *P. australis* can significantly differ in their morphology and growth (Clevering 1999; Vretare et al. 2001; Haldan et al. 2023). These differences may persist even after several years of growth under similar conditions, with notable variation in biomass production, morphology, carbon/nitrogen dynamics, and ontogeny (Kühl et al. 1999; Pauca-Comanescu et al. 1999; Rolletschek et al. 1999; Koppitz et al. 2000; Hansen et al. 2007; Eller and Brix 2012; Haldan et al. 2023; Kuprina et al. 2023).

In addition to its high genetic variation, populations of *P. australis* comprise multiple phylogenetic lineages that have also independently developed different ploidy levels ranging from 3 × to 12x, with tetraploid (4x) and octoploid (8x) cytotypes being the most prevalent (Clevering and Lissner 1999; Wang et al. 2024). Mixed cytotypes can also be found within a single population (Clevering and Lissner 1999; Lambertini et al. 2006; Hansen et al. 2007; Nakagawa et al. 2013).

While genetic and ploidy variations play an important role in plant adaptation, gene expression regulation and epigenetic modifications are the major contributors to phenotypic plasticity in plants, allowing them to respond to diverse environmental conditions and stresses (Grativol et al. 2012). Drought-induced gene expression varies widely in plants, and the precise molecular regulation under water deficit conditions remains poorly understood, especially for non-model species (Reddi et al*.* 2004). It has been shown that *P. australis* can tolerate drought stress through osmotic adjustment and improved water-use efficiency, maintaining stable chlorophyll content and photosynthesis rates even under severe drought conditions (Pagter et al. 2005; Liu et al. 2018). However, no data exist on molecular mechanisms such as gene regulation and DNA modifications in *P. australis* under drought stress.

This study provides novel insights into the transcriptomic and epigenetic changes underlying drought responses in *P. australis*. Furthermore, in conjunction with the growth and physiology study by Haldan et al. (2025), it presents the first comparative analysis of the drought response of the two most common ploidy levels (tetraploid and octoploid) of *P. australis*. To account for potential variation among genotypes from different origins, we used regional pairs of tetraploid and octoploid genotypes collected from three distinct geographical regions in a single mesocosm experiment. After exposing plants to drought of varying durations, we analyzed transcriptomic changes using RNA-seq and examined methylation patterns through methylation-sensitive amplification polymorphism (MSAP).

In this study, we hypothesize that drought influences gene expression and DNA methylation differently in tetraploids and octoploid *P. australis*. To test this, we address the following questions: (1) which genes and pathways are involved in the drought stress response? (2) How does the drought influence DNA methylation patterns? (3) Does ploidy level affect gene expression under non-stressed conditions? (4) Is there a difference in drought response between two ploidy levels?

## Materials and methods

### Experimental setup

To minimize the influence of geographic origin, we selected three regional genotype pairs representing two ploidy levels (tetraploid and octoploid) from distinct locations: Lake Fertő in Hungary (Hu4x and Hu8x), Lake Razim in Romania (Ro4x and Ro8x), and Sakhalin Island in Russia (Ru4x and Ru8x) (Table 1; precise coordinates are not available). The rhizome material of these genotypes was obtained in November 2019 from the live outdoor *P. australis* collection in the Aarhus University (Denmark, 56.228758 N, 10.127048 E), where the plants were growing in the common garden under similar conditions for more than 10 years. Ploidy level of the samples was proven by flow cytometry according to Kuprina et al. (2022).

**Table 1:** Sample metadata for *Phragmites australis* used in this study

Sample name	Days of drought	Ploidy	Genotype ID*	Group: ploidy	Group: treatment
c_Hu4x	0	Tetraploid	Pa 664 HU	4x	control
c_Hu8x	0	Octoploid	Pa 663 HU	8x	
c_Ro4x	0	Tetraploid	Pa 657 RO	4x	
c_Ro8x	0	Octoploid	Pa 659 RO	8x	
c_Ru4x	0	Tetraploid	Pa 205 RU	4x	
c_Ru8x	0	Octoploid	Pa 178 RU	8x	
2_Hu4x	20	Tetraploid	Pa 664 HU	4x	20d
2_Hu8x	20	Octoploid	Pa 663 HU	8x	
2_Ro4x	20	Tetraploid	Pa 657 RO	4x	
2_Ro8x	20	Octoploid	Pa 659 RO	8x	
2_Ru4x	20	Tetraploid	Pa 205 RU	4x	
2_Ru8x	20	Octoploid	Pa 178 RU	8x	
3_Hu4x	30	Tetraploid	Pa 664 HU	4x	30d
3_Hu8x	30	Octoploid	Pa 663 HU	8x	
3_Ro4x	30	Tetraploid	Pa 657 RO	4x	
3_Ro8x	30	Octoploid	Pa 659 RO	8x	
3_Ru4x	30	Tetraploid	Pa 205 RU	4x	
3_Ru8x	30	Octoploid	Pa 178 RU	8x	
4_Hu4x	40	Tetraploid	Pa 664 HU	4x	40d**
4_Hu8x	40	Octoploid	Pa 663 HU	8x	
4_Ro4x	40	Tetraploid	Pa 657 RO	4x	
4_Ro8x	40	Octoploid	Pa 659 RO	8x	
4_Ru4x	40	Tetraploid	Pa 205 RU	4x	
4_Ru8x	40	Octoploid	Pa 178 RU	8x	
5_Hu4x	50	Tetraploid	Pa 664 HU	4x	50d**
5_Hu8x	50	Octoploid	Pa 663 HU	8x	
5_Ro4x	50	Tetraploid	Pa 657 RO	4x	
5_Ro8x	50	Octoploid	Pa 659 RO	8x	
5_Ru4x	50	Tetraploid	Pa 205 RU	4x	
5_Ru8x	50	Octoploid	Pa 178 RU	8x	

^*^Lambertini et al. 2006; **Included only in MSAP

Meristematic propagation of rhizome buds was carried out at the Julius Kühn Institute in Braunschweig, Germany. The resulting plantlets were then transplanted to the Arboretum of Greifswald University (Germany, 54.092063 N, 13.410133 E) in June 2020.

Plants were grown in plastic tubes (20 cm diameter, 60 cm height) filled with a 2:1 mixture of white peat (pH 6; Rostocker Humus & Erden GmbH, Rostock, Germany) and sand. Tubes were sealed at the bottom with water-permeable root fleece (polypropylene, 150 g/m^2^). Two clones per tube were planted on 8 June 2020 and received 0.744 g of slow-release fertilizer (Basacote Plus 6 M: 16 N + 8 P + 12 K (+ 2 Mg + 5 S) + trace elements; Compo Expert, Münster, Germany). Beginning on 9 April 2021, plants were fertilized weekly for seven weeks with 15 g of Hakaphos Blau (15N + 10P + 15 K + 2 Mg + trace elements; Compo Expert), and, from the second fertilization onwards, additionally with 0.5 g iron chelate (YaraTera TENSO IRON 58: 6% water soluble iron, 4% HBED iron chelate, 1.8% EDDHMA iron chelate, YARA GmbH & Co. KG, Dülmen, Germany) dissolved in 0.25 L tap water per tube.

**Fig. 1:**
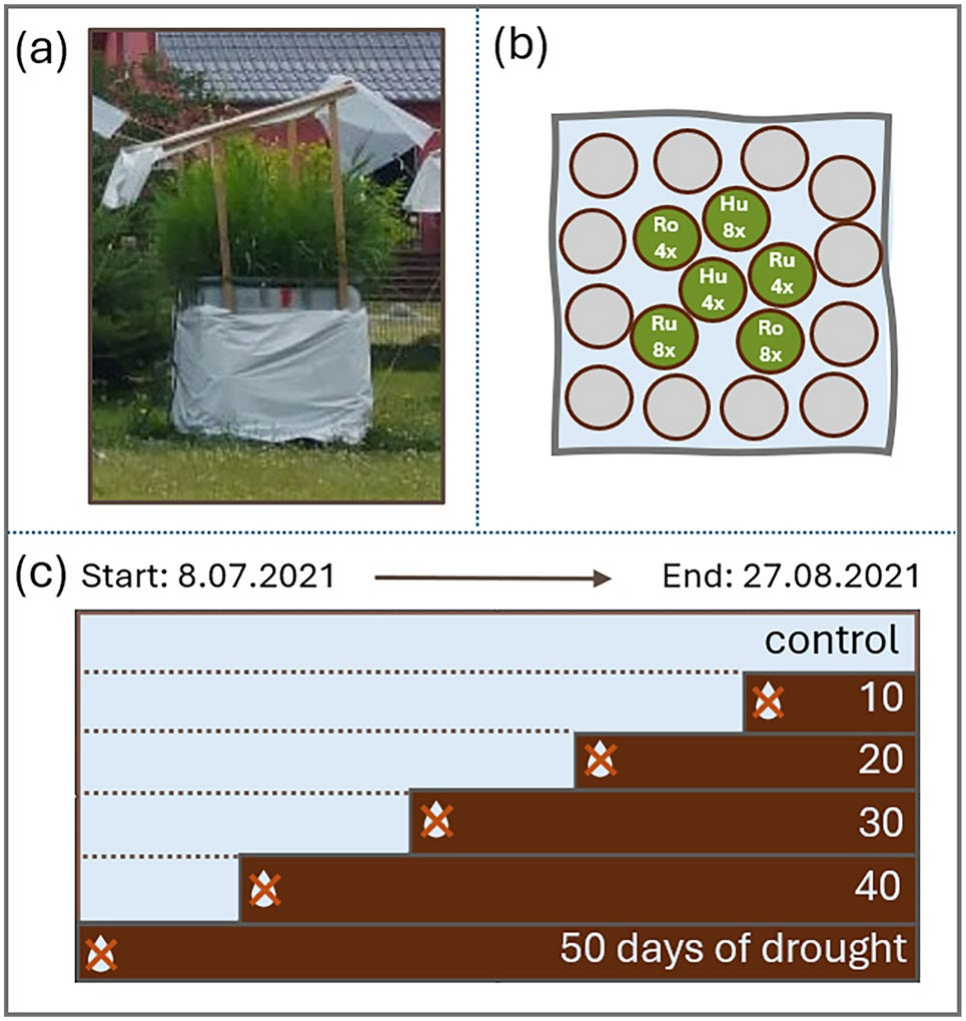
Setup of the mesocosm drought experiment: **a** container representing one of the six drought treatment levels; **b** example arrangement of tubes containing six different genotypes of experimental *Phragmites australis* plants (one per tube; labeled green circles) and edge-effect control plants (empty grey circles); **c** water removal schedule for six drought treatment levels

Tubes with the plants were placed in a 1 × 1 × 1 m container, which was filled with communal tap water up to the soil surface and covered with a roof made of greenhouse foil (Lumisol PGH-UV5/UV-B open, UV-B permeable, light permeability 88%, Poppen Gewächhausbau, Edewecht-Jeddeloh, Germany) (Fig. 1a). Containers, each representing a treatment level, were arranged in a quasi-random order without shading each other. One tube per genotype inside the container was arranged quasi-randomly and surrounded by 12 tubes with *P. australis* plants to avoid edge effects, on which response parameters were not quantified (Fig. 1b). Three soil moisture sensors (TMS-4, TOMST s.r.o., Prague, Czech Republic) were installed in the tubes of edge-positioned plants in each container.

Subsequently, the plants were exposed to a mesocosm gradient experiment comprising six treatment levels, ranging from 0 to 50 days of drought in 10-day increments. Starting on 8 May 2021, water was drained every 10 days from a respective container so that the drought ended at the same date for all drought levels (Fig. 1c). The experiment ended on 27 August 2021. Volumetric soil moisture was 0.52 in the control, 0.12 after 10 days of drought, 0.07 after 20 days, 0.04 after 30 days, and 0.03 after both 40 and 50 days. The permanent wilting point (5.2%) was reached, on average 24 days after drainage. On the final day of the experiment, between 9:00 and 13:00, material was collected for transcriptomic and epigenetic analyses.

For transcriptome analysis, three levels of drought treatment were used: 0 days (control), 20, and 30 days of drought. The 20- and 30-day treatment durations were selected as they fall below and above the permanent wilting point, respectively. Leaves from the longer drought treatments exhibited yellowing and were excluded from analysis due to the potential risk of RNA degradation. In each treatment level, the same six different genotypes were studied, giving six biological replicates and 18 samples in total (Table 1). For each sample, tissue from three leaves was taken from each of three stems growing in one tube; a 0.5 cm^2^ piece of a second or third from the top fully developed and visually healthy leaf was cut in the middle position from the leaf base. Thus, each biological replicate consisted of pooled leaf material from three stems of a single genotype. The leaf samples were incubated overnight at + 6 °C in RNAlater solution (Thermo Scientific, Waltham, MA, USA) and then stored at − 20 °C for 1 month.

For epigenetic analysis, six drought treatment levels were used: 0 days (control), 10, 20, 30, 40, and 50 days of drought, yielding six biological replicates per treatment and 36 samples in total. One leaf per plant was collected from three different stems and stored in a sealed plastic bag with silica gel for preservation.

### RNA sequencing

Leaf material from one sample was pooled and homogenized in liquid nitrogen with mortar and pestle, then its total RNA was extracted using the innuPrep Plant RNA Kit (Analytik Jena, Jena, Germany) according to the manufacturer’s protocol. Directly after its extraction, RNA was examined for its concentration, purity, and integrity using NanoDrop Lite spectrophotometer (Thermo Scientific) and a 1.5% (w/v) agarose gel. Then RNA was subjected to DNase treatment using DNase I (Thermo Scientific) according to the manufacturer’s protocol and placed at − 80 °C. Samples were shipped on dry ice to Eurofins Genomics (Konstanz, Germany) for the strand-specific cDNA library preparation and following sequencing on Illumina NovaSeq 6000 (Illumina Inc., San Diego, CA, USA) in S4 PE150 XP mode.

One RNA sample (c_Ro4x) was additionally sequenced using MinION Mk1C Model MIN-101C with Flow Cell (R10.4.1) and Direct RNA Sequencing Kit (SQK-RNA002) according to the manufacturer’s protocol (all from Oxford Nanopore, Oxford, UK).

### Transcriptomic data pre-processing

All bioinformatic steps were conducted using conda 23.9.0 and python 3.9.16.

Ribosomal RNA was filtered out from the raw Illumina reads using the program SortMeRNA 4.3.4 (Kopylova et al. 2012). Then the reads were trimmed for Illumina adapters, and low-quality bases and the low-quality reads were discarded using trimmomatic 0.33 (Bolger et al. 2014) with the following parameters: *ILLUMINACLIP:TruSeq3-PE.fa:2:30:10, SLIDINGWINDOW:5:30, MINLEN:100.* Illumina and Nanopore reads were taxonomically classified using the Kraken2 tool (Wood et al. 2019) and the KrakenTools 1.2 suite (Lu et al. 2022). Sequence databases used for classification included Standard (covering archaea, bacteria, viruses, plasmids, human, and vectors) and PlusPFP-16 (including protozoa and fungi), both released on 09-04-2024. Additional filtering of contamination was conducted by the NCBI Foreign Contamination Screen tool (Astashyn et al. 2024). Quality of the resulted reads was evaluated using programs FastQC 0.12.0 (Andrews 2010), MultiQC 1.14, and NanoPlot 1.43.0 (Ewels et al. 2016; De Coster and Rademakers 2023).

### Assembly, annotation, and quantification of transcripts

A flowchart of the data analysis workflow, scripts, and MultiQC report for clean reads is available on GitHub: https://github.com/kuprinak/Phragmites_drought_RNAseq.

A hybrid de novo transcriptome assembly was performed in rnaSPAdes 3.15.4 (Prjibelski et al. 2020) using raw short reads of all control samples, as well as raw long reads of the sample c_Ro4x. The resulting transcriptome was analyzed using rnaQUAST 2.2.3 (Bushmanova et al. 2016) to calculate basic statistics and predict gene numbers based on the GeneMarkS-T database. In addition, the completeness of the transcriptome was assessed with BUSCO 5.4.4 (Manni et al. 2021) in the *euk_tran* mode, using the *poales_odb10* lineage dataset (creation date: 08–01-2024). Annotation of the transcriptome was conducted using InterProScan 5.72–103.0 (Jones et al. 2014) against Pfam and SUPERFAMILY databases. Functional annotation has been done in KAAS 2.1 (Kyoto Encyclopedia of Genes and Genomes Automatic (KEGG) Annotation Server) (Moriya et al. 2007) using BLAST search against gene datasets of *Oryza sativa* subsp*. japonica*, *Aegilops tauschii*, and *Zea mays*.

Quantification of reads was performed in Salmon 1.10.3 (Patro et al. 2017) via mapping clean short reads against assembled transcriptome with the following parameters: -*l ISF, -gcBias, -seqBias, -posBias, -validateMappings*.

### Differential expression analysis

Analysis and visualization of differential expression of the transcripts was conducted in R 4.4.2 (R Core Team 2024) using RStudio 2023.12.1.402 (RStudio Team 2020). Results of the read quantification were imported to the R environment using *tximport* 1.32.0 package. The raw read counts were examined for variance distribution by plotting the variance against the mean count for each transcript. Raw read counts were then normalized using median of ratios method provided by the *DESeq2* 1.44.0 package (Love et al. 2014). To conduct a principal component analysis (PCA), transformed read counts were additionally “regularized” via log transformation (function “rlog”), and then 300 transcripts with the highest variation were used for the analysis (function “plotPCA*”*). The matrix of the counts was used to draw a clustered heatmap in the package *pheatmap* 1.0.12 (Kolde 2018).

Transformed read counts were used for the calculation of Log2 fold changes (Log2FC) of the transcript abundances between groups of samples (Table 1) using the package *DESeq2*. To reduce the impact of random noise and control the false discovery rate, the resulted Log2FC were shrunk towards zero using method “ashr”. The transcripts with absolute Log2FC higher than 1.2 were assigned to differentially expressed genes (DEGs). The results were visualized using R packages *ggplot2 3.5.1*, *ggrepel* 0.9.6, and *ggvenn* 0.1.10 (Wickham 2016; Slowikowski 2024).

The enrichment analysis was conducted for DEGs grouped by assigned KEGG functions and Gene Ontology (GO) terms (biological processes, molecular functions and cellular components) using *clusterProfiler* 4.12.6 R package (Xu et al. 2024). The list of InterPro entries assigned to the GO terms was extracted from the Gene Ontology Resource webpage: https://current.geneontology.org/ontology/external2go/interpro2go (version date: 2024/04/08) (Mitchell et al. 2015). False discovery rate (FDR) adjustment was performed with *p *value and q value cutoffs set at 0.05.

The script and the output results of the data analysis in R can be found on GitHub: https://github.com/kuprinak/Phragmites_drought_RNAseq.

### Molecular analysis of MSAP

Various epigenetic mechanisms influence gene expression, but DNA methylation has been the primary focus of ecological studies (Schrey et al. 2013). Here, we employ a modified methylation-sensitive amplification polymorphism (MSAP) protocol to assess variation in DNA methylation at multiple sites of the genome (Reyna-López et al. 1997).

Total DNA was extracted from silica dry leaf material using the Mag-Bind Plant DNA DS Kit (OMEGA BioTek, Norcross, USA) according to the manufacturer’s protocol with the help of the KingFischer Flex Purification System (ThermoFisher, Waltham, USA). The concentration, purity, and integrity of extracted DNA were measured by NanoDrop Lite spectrophotometer (Thermo Scientific, USA) and via electrophoresis on a 1.5% (w/v) agarose gel. Four samples (Ru4x from control, Hu4x from 40 days of drought, Ro4x and Ro8x from 50 days of drought) did not yield sufficient DNA (1 µg). Consequently, 32 samples of the original 36 were used for the downstream analyses.

To control the error rate, seven samples were processed in two technical replicates (starting with DNA digestion).

DNA samples were digested separately using two restriction enzyme sets, each consisting of methylation-insensitive EcoRI and either methylation-sensitive MspI or HpaII. While EcoRI cuts its target site regardless of methylation of cytosine, MspI does not cut when the inner cytosine is methylated, and HpaII does not cut when either or both cytosines are fully or hemi-methylated (Roberts et al*.* 2007). Each 50 µL reaction contained 500 ng DNA, ddH₂O, 5 µL 10 × Cut-Smart Buffer, 5 U EcoRI, and either 10 U MspI or 5 U HpaII (all from New England Biolabs (NEB), Ipswich, MA, USA). Reaction mixtures were incubated for 3 h at 37 °C.

Resulted DNA fragments were ligated with the adaptors (EcoRI: 5’-CTCGTAGACTGCGTACCAATTGGTACGCAGTC-3’ and HpaII/MspI: 5’-GACGATGAGTCTCGATCGATCGAGACTCAT-3’). Ligation mix of a total volume of 40 µl contained 20 µl restriction product, 4 µl 10 × T4 ligation buffer (NEB), 3.3 U T4 ligase (NEB), 0.5 mM ATP (NEB), 40 pmol EcoRI-adaptor, and 200 pmol HpaII/MspI-adaptor and ddH₂O. The mixtures were incubated for 8 h at 16 °C. The ligation product was then diluted sixfold with ddH₂O.

Pre-selective amplification was conducted in two steps: first, 3.5 µL of diluted ligation product was mixed with 1 µL 10 × PCR buffer, 0.88 µL 25 mM MgCl_2_, 0.25 µL 10 mM dNTPs (all from Axon Labortechnik, Kaiserslautern, Germany), 3.15 µL ddH₂O, and 0.07 µL 5U/µL MolTaq DNA polymerase (Goffin Molecular Technologies, Beesd, Netherlands). The mix was incubated for 10 min at 60 °C. After this step, 0.3 µL 10 mM EcoRI-A primer (5’-GACTGCGTACCAATTCA-3’), 0.3 µL 10 mM HpaII/MspI-A primer (5’-GATGAGTCCTGATCGGA-3’), 0.25 µL 10 mM dNTPs, 0.07 µL 5U/µL MolTaq DNA polymerase, and 0.07 µL ddH₂O were added. The amplification was conducted with SensoQuest thermocycler (Göttingen, Germany) with the following program: (1) 2 min 30 s at 94 °C; (2) 10 cycles of amplification consisting of 30 s at 94 °C, 30 s at 66—56 °C (touch-down with − 1 °C per cycle) and 2 min at 72 °C; (3) 20 cycles of amplification with 30 s at 94 °C, 30 s at 56 °C, and 2 min at 72 °C; 3 min final extension at 72 °C. The resulting product was diluted 20-fold with ddH₂O.

Selective amplification was conducted with two sets of primers separately: (1) EcoRI-AGA with HpaII/MspI-ATG and (2) EcoRI-ACT with HpaII/MspI-AGT. Primers had a FAM fluorochrome tag on the 5′ tail. Each 10 µL reaction mixture consisted of 0.9 µL of diluted pre-selective amplification product, 6.04 µL ddH_2_0, 1 µL 10 × PCR buffer, 2.2 mM MgCl_2_, 0.5 mM dNTPs, 450 pmol EcoRI primer, and 160 pmol HpaII/MspI primer. Amplification conditions were the same as in the pre-selective amplification.

The samples were prepared for capillary electrophoresis by adding 1.2 μl PCR product to 11.85 μl HiDi^™^ Formamide, 0.15 μl GeneScan^™^ 500 LIZ^™^ Size Standard, and denaturation for 5 min at 95 °C. Capillary electrophoresis was conducted with an ABI 3130XL Genetic Analyser. All were obtained from Applied Biosystems (Foster City, CA, USA).

### Data analysis of MSAP

Detection and sizing of the peaks in resulted electropherograms were conducted via the program PeakScanner 2.0 (Applied Biosystems) with the following settings: full size range, minimum peak height of 50, light peak smoothing, and “local Southern” size calling method. Output peak size table was further processed using R package *RawGeno* 2.0–2 (Arrigo et al. 2012) and R 3.6.2. Binning was conducted manually; peaks were scored in a range from 60 to 500 bp with the lowest fluorescence of 300 RFU. Error rates were calculated for each set of restriction enzymes, after which technical replicates were excluded from further analysis. A single combined 0/1 matrix of distance was created for both primer pairs and restriction enzyme set.

The resulting matrix was analyzed using the R package *msap* 1.1.8 (Pérez-Figueroa et al. 2013) in R 4.2.2. The package compared fragment presence between enzyme pairs (MspI vs. HpaII) to assess the methylation sensitivity of the corresponding restriction sites. It then estimated methylation levels across sample groups (ploidy levels and treatments), categorizing them as: (1) unmethylated, (2) hemi-methylated, (3) methylated at the internal cytosine, or (4) fully methylated (or absent target site). Pearson’s correlation was used to assess the relationship between drought duration and the levels of unmethylation or full methylation. In addition, molecular variance analysis (AMOVA) with 10,000 permutations and principal coordinate analysis (PCoA) based on Euclidean distances were conducted using the *msap* package. The script and the output results of the data analysis in R can be found on GitHub: https://github.com/kuprinak/Phragmites_drought_RNAseq.

## Results

### RNA library

Illumina sequencing produced a total of 1,037.1 M raw read pairs with the average sequencing depth of 60 × per sample (Table 2). After trimming and filtering out rRNA and contamination, 690.0 M clean read pairs were left (average ± SD: 38.3 ± 18.5 M read pairs; Table 2). All sample libraries successfully passed the assessments of Phred read quality, per-base N content, adaptors, and overrepresented sequences content.

**Table 2:** Sequencing metrics for transcriptomic analysis

Sample name	Number of raw read (million pairs)	Number of clean read (million pairs)	Mapping rate, %	Sequencing depth, x
c_Hu4x	59.06	39.32	75.69	62
c_Hu8x	52.59	40.18	74.64	55
c_Ro4x	60.61	47.03	74.64	64
c_Ro8x	56.02	35.00	75.49	59
c_Ru4x	40.85	26.61	74.98	43
c_Ru8x	56.38	43.83	74.10	59
2_Hu4x	14.79	9.39	72.63	16
2_Hu8x	70.85	54.81	74.80	74
2_Ro4x	91.54	69.09	72.33	96
2_Ro8x	40.73	29.45	68.11	43
2_Ru4x	56.61	44.63	73.18	59
2_Ru8x	49.78	37.24	72.88	52
3_Hu4x	31.88	20.52	73.18	33
3_Hu8x	16.63	11.01	74.10	17
3_Ro4x	116.42	110.08	73.74	122
3_Ro8x	56.76	43.28	73.82	60
3_Ru4x	97.18	75.00	74.65	102
3_Ru8x	68.34	52.63	73.01	72

Nanopore sequencing of the sample c_Ro4x generated 618.3 K reads with N50 length of 1.35 kb. After the removal of contaminating reads (mostly comprising DNA of Saccharomycetes), 273,336 reads were left (N50 read length = 918 bp, the longest read = 132 Kb).

### Transcriptome

The assembled transcriptome comprised 258.76 Mb across 191,527 sequences (transcripts), with an N50 length of 2,279 bp. BUSCO estimated the transcriptome completeness at 90.7%, while rnaQUAST predicted 88,348 genes. InterProScan successfully annotated 93,050 transcripts, with 92.5% assigned to 5,652 unique InterPro entries. In addition, all transcripts were mapped to either one of 5,218 unique Pfam entries or 1,095 unique SUPERFAMILY entries. Functional annotation identified 21% of the transcripts as associated with 750 distinct KEGG identifiers.

The MultiQC reports of the quality of raw and clean reads can be found on GitHub: https://github.com/kuprinak/Phragmites_drought_RNAseq. Clean short and long reads, as well as assembled transcriptome, can be found on NCBI (PRJNA1195549).

### Differential gene expression

**Fig. 2:**
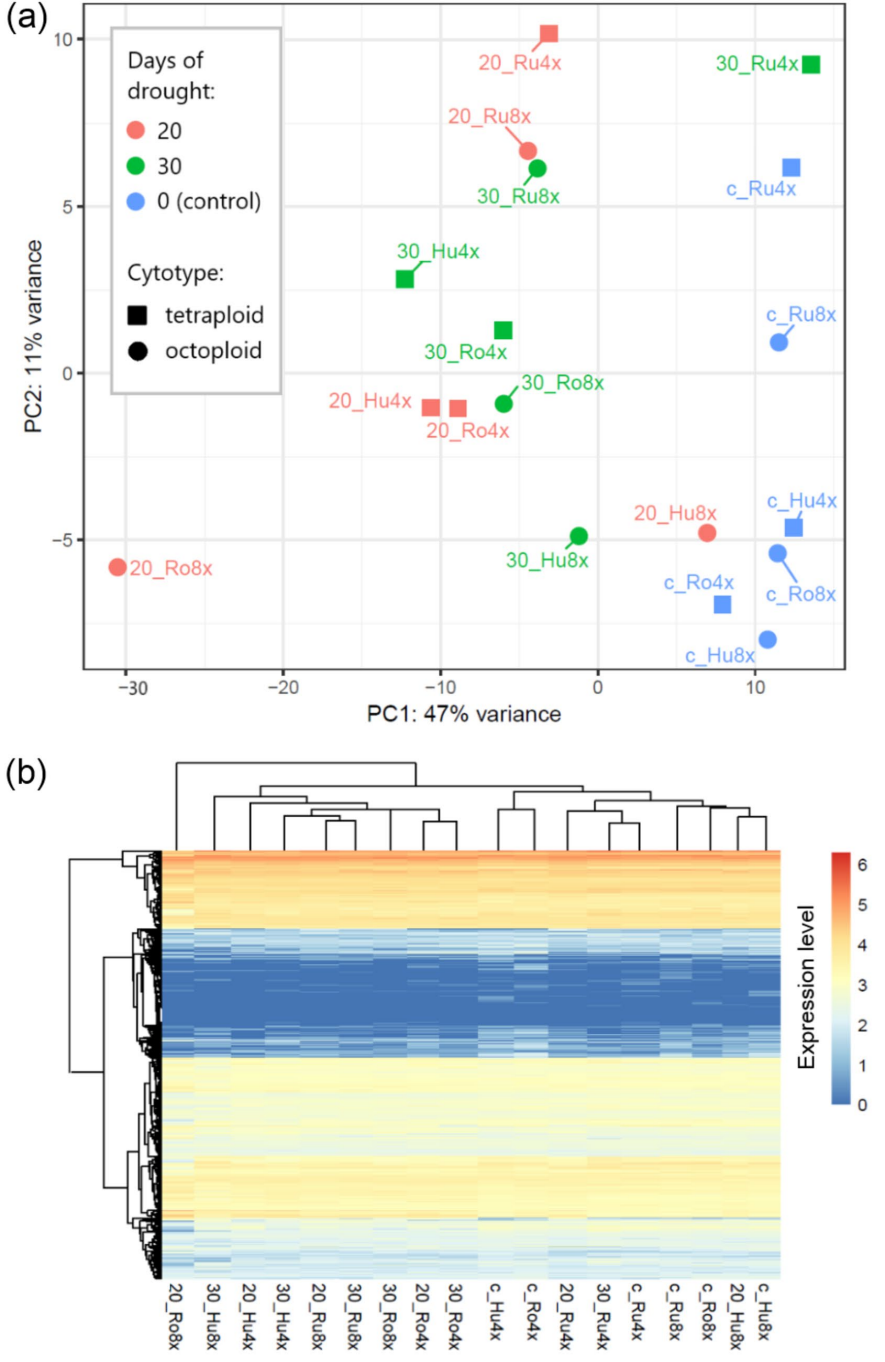
**a** Ordination diagram of PCA of normalized counts of 300 most variable transcripts. **b** Heatmap and hierarchical clustering of gene expression based on log10-transformed normalized reads counts. Transcriptomic data were obtained for six genotypes of *Phragmites australis*, representing two cytotypes (tetraploid and octoploid) and three geographic origins (Hu, Ro, Ru), each subjected to drought treatments of 0, 20, or 30 days

Mapping of the clean reads on the transcriptome resulted in an average of 73.66% mapping rate (Table 2). PCA showed that the first component axis explains 47% of the variance and the second 11% (Fig. 2a). Samples along the first axis were separated into two clusters: control (with 30_Ru4x and 20_Hu8x) and treated samples. Along the second axis, samples were separated by origin: from Europe (Hu and Ro) and East Asia (Ru). The heatmap of gene expression with hierarchical clustering revealed a pattern consistent with the first principal component axis in the PCA (Fig. 2b).

**Fig. 3:**
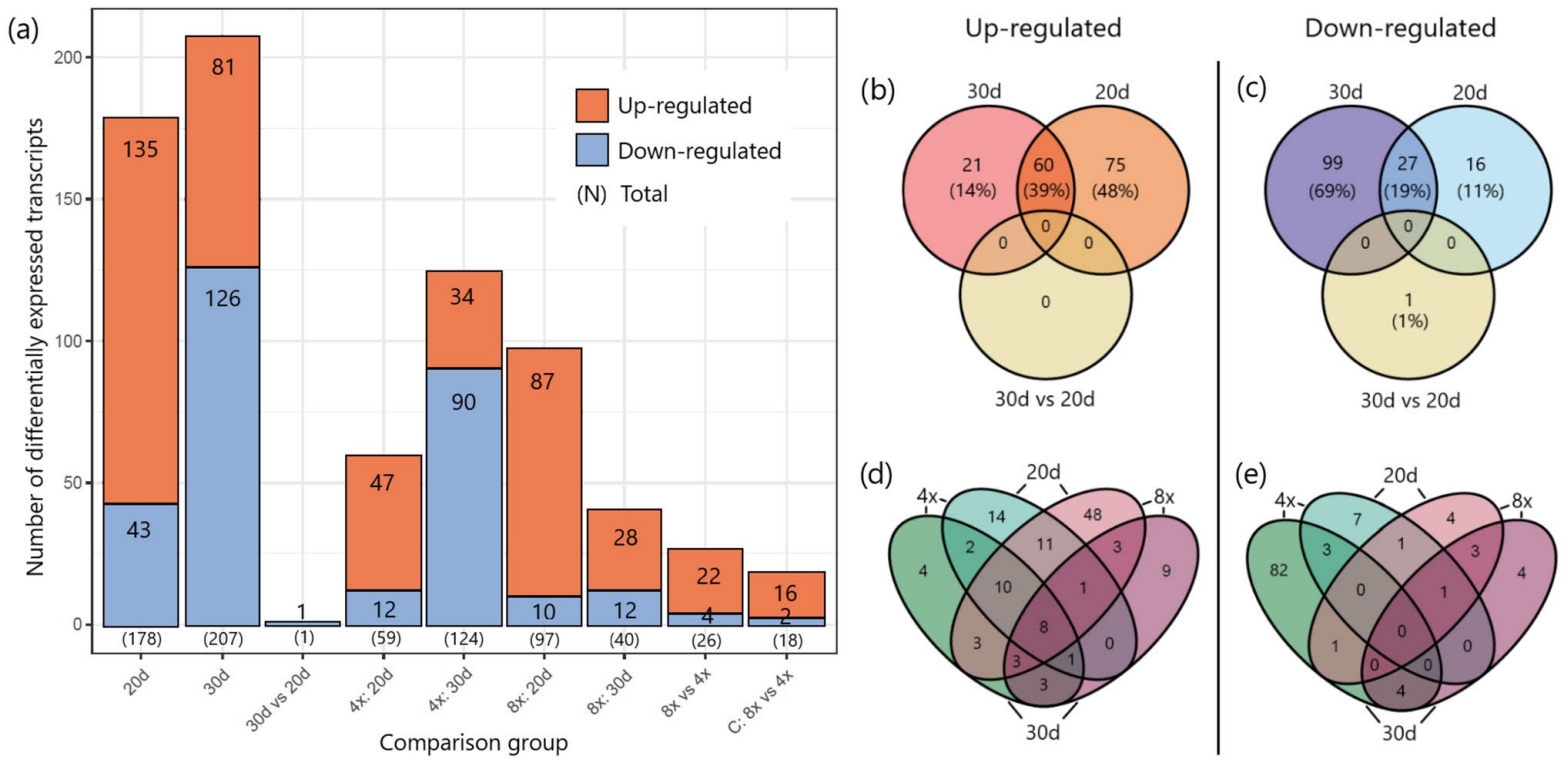
**a** Total numbers of differentially expressed genes across nine comparisons. **b**–**e** Venn diagrams displaying unique and shared **b**, **d** upregulated and **c**, **e** downregulated genes, with **b**, **c** showing all ploidy levels and (**d**, **e**) focusing on comparisons between tetraploid (4x) and octoploid (8x) samples separately. Transcriptomic analyses were conducted on leaf material of six genotypes (biological replicates) of *Phragmites australis*, comprising three tetraploid and three octoploid cytotypes. Samples of each genotype were subjected to drought treatments of 0 days (control), 20 days (20d), and 30 days (30d). Differential gene expression analysis was performed on 4788 transcripts

Differential gene expression analysis was conducted using 4,788 transcripts. The full list of Log2FC, *p *values, and adjusted *p *values for each transcript, and each comparison can be found in Suppl. Table 1. The number of DEGs varied across the comparisons of sample groups. After 20 days of drought treatment, 135 genes were upregulated, and 43 were downregulated compared to the control samples (Fig. 3a). By the 30th day of drought, 81 genes were upregulated and 126 were downregulated (Fig. 3a). Between the 20- and 30-day drought treatments, 60 (39%) upregulated and 27 (19%) downregulated genes were shared (Fig. 2b, c). Only one downregulated gene and no upregulated genes were identified when comparing samples from 30 days to those from 20 days of drought.

**Fig. 4:**
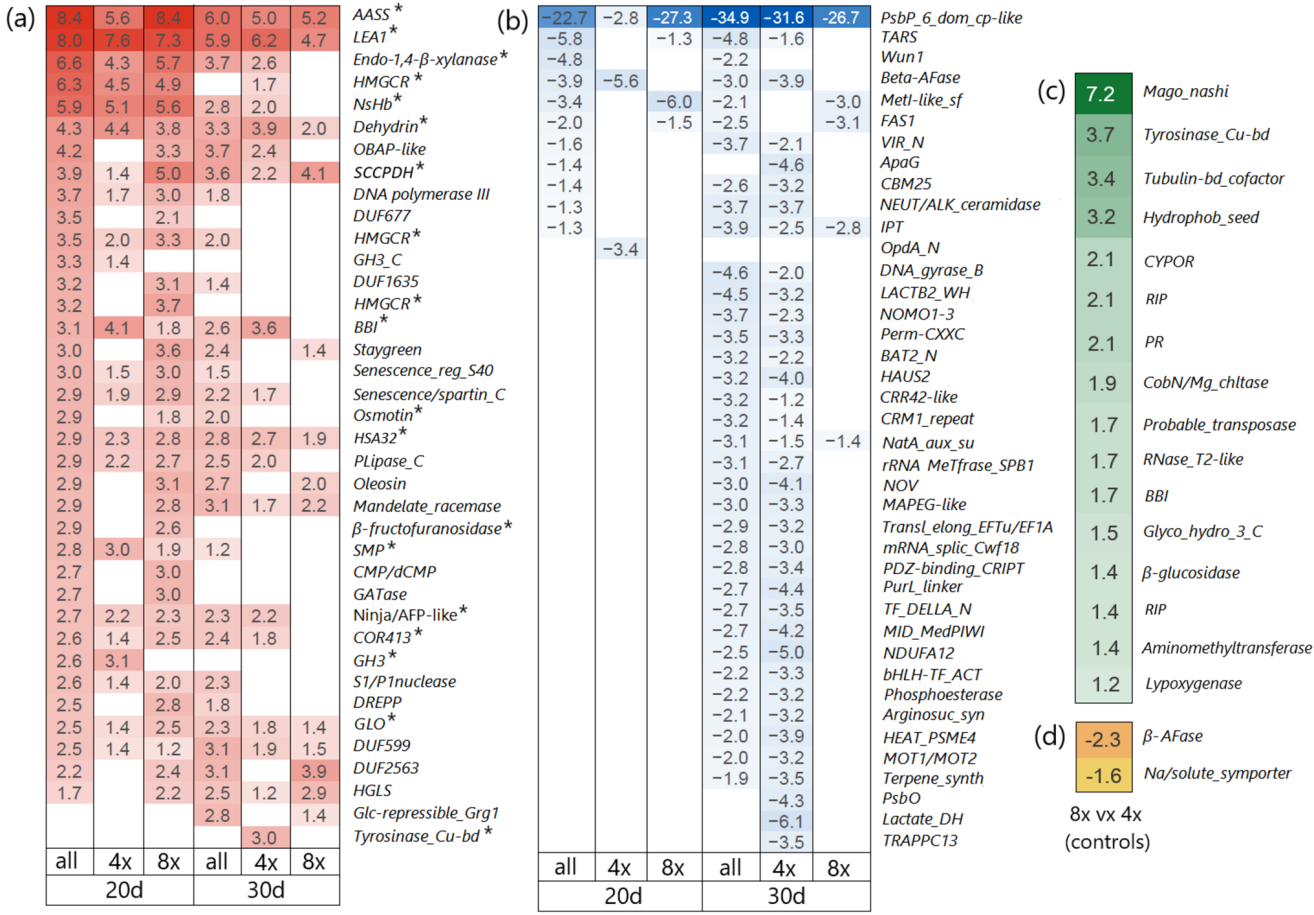
Heatmaps with the **a** upregulated genes with Log2 fold change > 2.0 and with **b** downregulated transcripts with Log2 fold change < − 3.0 in *Phragmites australis* leaf samples after 20 days (*n* = 6) and 30 days (*n* = 6) of drought compared to corresponding control samples (zero days of drought, *n* = 6). Heatmaps **c** with all upregulated and **d** all downregulated transcripts identified in control octoploid samples (8x, *n* = 3) compared to control tetraploid samples (4x, *n* = 3). Numbers represent Log2 fold changes for genes with significant adjusted *p* values (*α* = 0.05). Differential gene expression analyses were performed on 4,788 transcripts. *genes putatively involved in the drought response

Among all DEGs, eight genes were upregulated in both drought treatments and both ploidy levels. They encoded: α-aminoadipic semialdehyde synthase (*AASS*; IPR007886), late embryogenesis abundant protein 1 (*LEA1*; IPR005513), dehydrin (IPR000167), saccharopine dehydrogenase (*SCCPHD*; IPR032095), heat shock-associated protein 32 (*HSA32*; IPR003830), heparan-α-glucosaminide N-acetyltransferase (*HGSNAT;* IPR012429), glyoxalase (*GLO*; IPR029068)*,* and domain of unknown function 599 (*DUF599*; IPR006747)*.* Among these eight genes, seven showed Log2FC > 2 in at least one comparison (Fig. 4a). The BLASTN for three upregulated genes with unknown function *DUF599*, *DUF677*, and *DUF163* found the closest match with *P. australis* uncharacterized mRNA.

No genes were consistently downregulated across both drought treatments and ploidy levels. The most highly downregulated gene after both 20 and 30 days of drought encoded the domain of unknown function (*DUF3007* = IPR021562, up to 34.9-fold) (Fig. 4b). BLASTN search identified its closest match with the psbP domain-containing protein 6, chloroplastic-like (LOC133908951) of *P. australis*.

A distinct pattern of transcripts’ expression appeared when comparing samples with different ploidy levels (Fig. 3a). Tetraploid samples exhibited more pronounced expression changes on the 30th day of drought, with 59 upregulated and 124 downregulated DEGs, whereas octoploid samples showed greater expression changes on the 20th day, with 97 upregulated and 40 downregulated DEGs. Tetraploid samples had the highest number of unique downregulated transcripts (89), whereas octoploid samples exhibited the highest number of unique upregulated transcripts (57) under drought stress (Fig. 3d, e).

The comparison of octoploid and tetraploid controls revealed 16 upregulated transcripts and 2 downregulated transcripts in octoploid samples (Fig. 4a, c, d). The most highly upregulated genes (with the Log2FC > 2) encoded Mago Nashi protein (IPR004023, 7.2-fold), tyrosinase (Cu-binding domain, IPR002227, 3.7-fold), tubulin binding cofactor (IPR012945, 3.4-fold), hydrophobic seed protein domain (IPR027923, 3.2-fold), NADPH-cytochrome P450 oxidoreductase (CYPOR, IPR003097, 2.1-fold), ribosome-inactivating protein (RIP, IPR036041, 2.1-fold), and pathogenesis-related protein (PR, IPR014044, 2.1-fold). Two downregulated genes encoded β-L-arabinofuranosidase (β-AFase, IPR012878, 2.3-fold) and sodium/solute symporter (IPR001734, 1.6-fold).

### Enrichment analysis

**Fig. 5:**
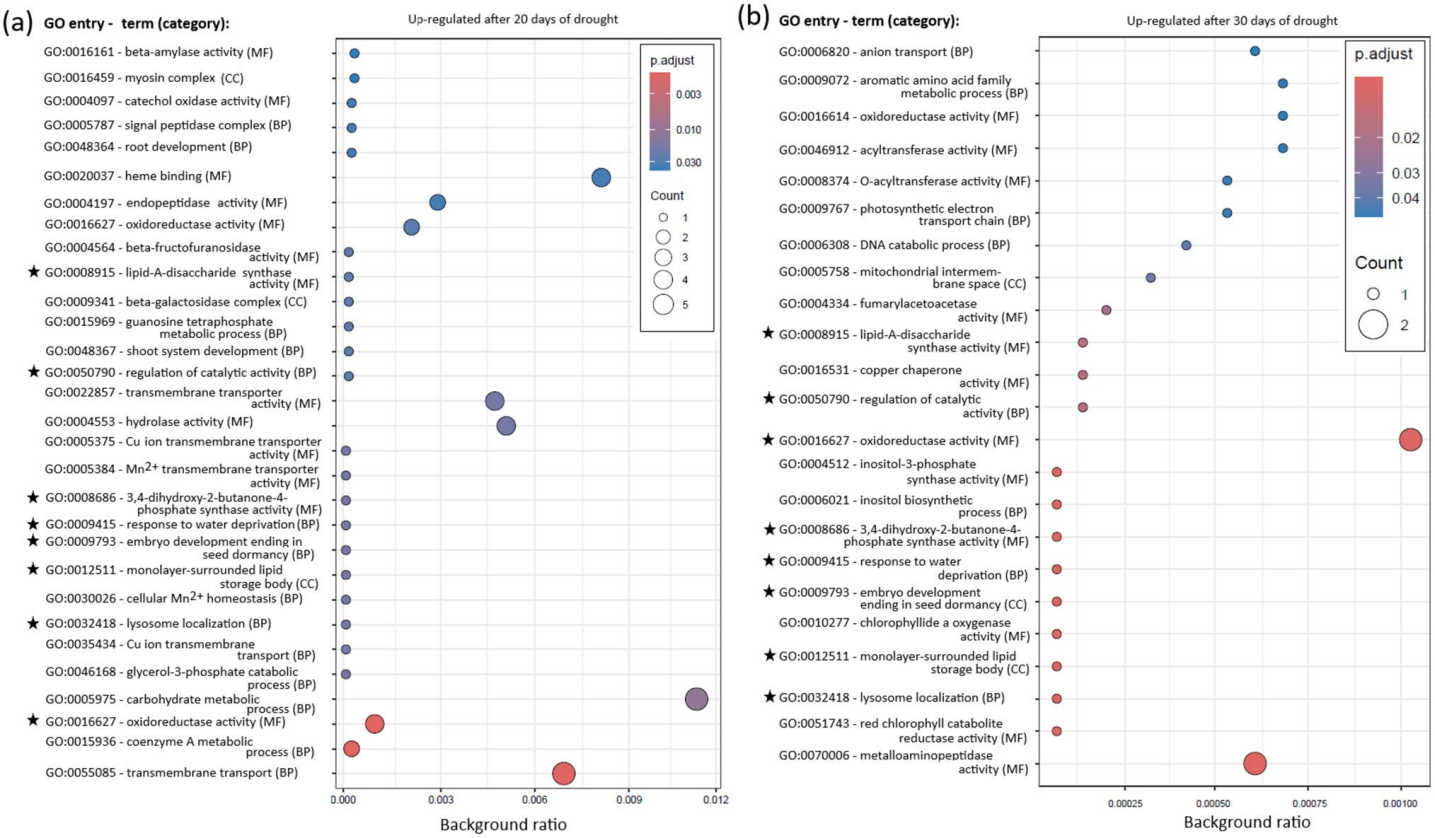
Enriched Gene Ontology (GO) terms upregulated after **a** 20 days and **b** 30 days of drought in leaf samples of six *Phragmites australis* genotypes, compared to control samples (no drought, same genotypes). GO categories: *BP* biological process, *CC* cellular component, *MF* molecular function. Asterisks indicate GO terms upregulated and enriched after both 20 and 30 days of drought. Counts represent the number of transcripts assigned to the corresponding term. Background ratio quantifies the proportion of transcripts in a GO term relative to all analyzed transcripts

**Fig. 6:**
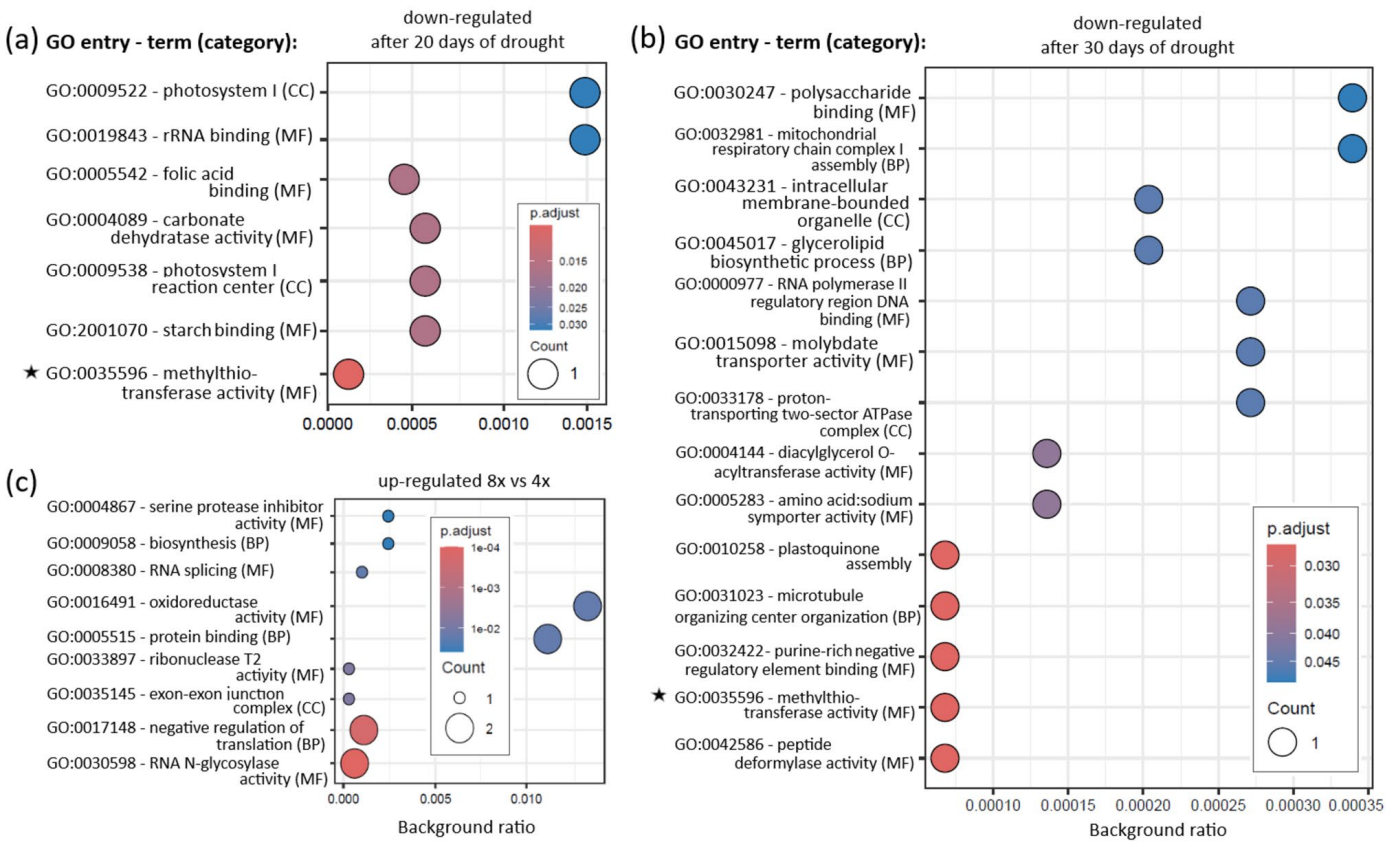
Enriched Gene Ontology (GO) terms downregulated after **a** 20 days and **b** 30 days of drought in leaf samples of six *Phragmites australis* genotypes, compared to control samples (no drought, same genotypes). **c** GO terms upregulated in leaf samples of three octoploid *Phragmites australis* genotypes compared to three tetraploid genotypes (no drought, same genotypes). GO categories: *BP* biological process, *CC* cellular component, *MF* molecular function. Asterisks indicate GO terms downregulated and enriched after both 20 and 30 days of drought. Counts represent the number of transcripts assigned to the corresponding term. Background ratio quantifies the proportion of transcripts in a GO term relative to all analyzed transcripts

Eight GO entries were consistently enriched in the samples after 20 and 30 days of drought: seven were linked to upregulated transcripts (Fig. 5a, b) and one to downregulated transcripts (Fig. 6a, b). For KEGG enrichment analysis, three genes were upregulated after both 20 and 30 days of drought: *cathepsin B* (K01363, involved in lysis), *acetyl-CoA synthetase* (K01895, associated with glycolysis/gluconeogenesis, pyruvate metabolism, glyoxylate and dicarboxylate metabolism, and propanoate metabolism), and *PPIP5K1* (K13024, involved in the phosphatidylinositol signaling system within environmental information processing). The full list of enriched GO and KEGG entries can be found in Supplementary File 1.

The comparison of octoploid control samples with tetraploid control samples revealed nine upregulated GO entries (Fig. 6c) and one upregulated KEGG entry, *lipoxygenase* (K00454), involved in the linoleic acid metabolism pathway.

### MSAP

As a result, 32 samples in 252 loci were screened for DNA methylation patterns. The detected error rate was 8.3% for HpaII and 7.0% for MspI in primer set 1, and 4.7% for HpaII and 6.7% for MspI in primer set 2.

**Fig. 7:**
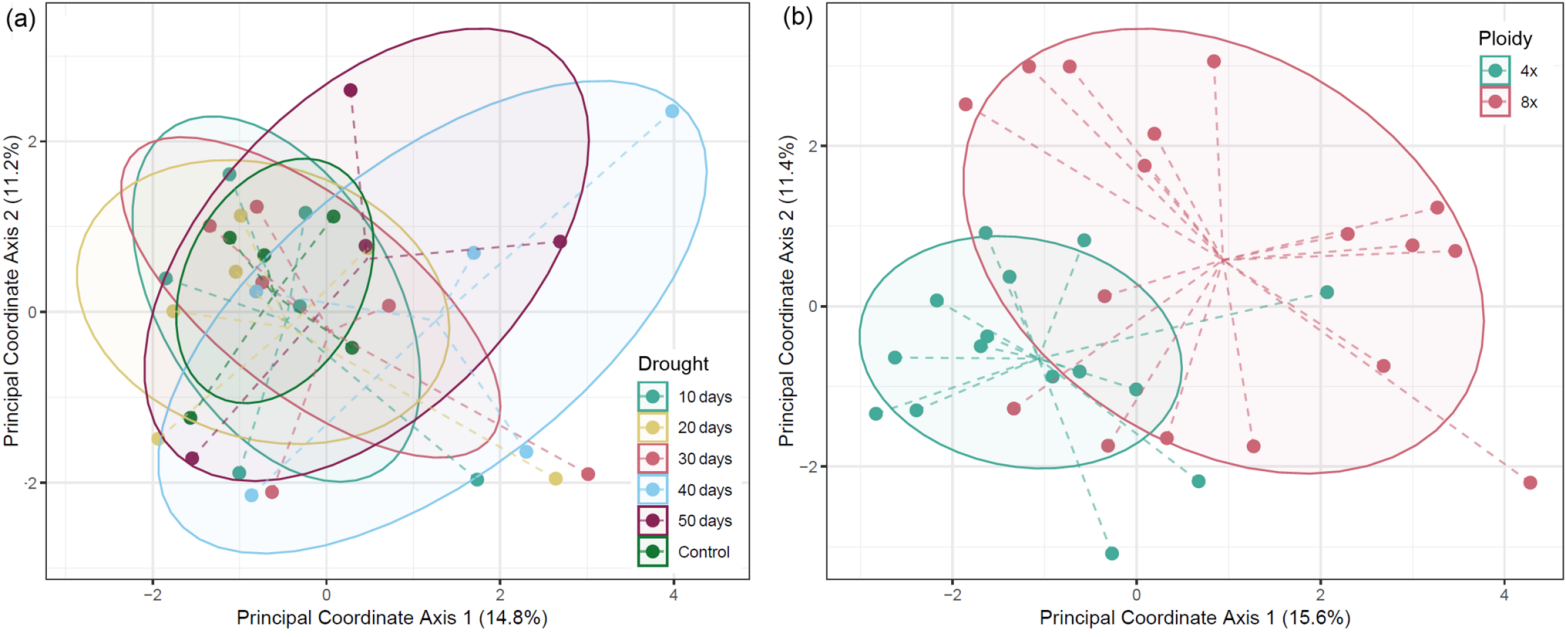
Ordination diagram of PCoA calculated on 248 methylation-sensitive loci for 32 leaf samples of *Phragmites australis* **a** under 6 different drought treatments and **b** with 2 ploidy levels. The same six genotypes were included in each treatment level (except for Ru4x from control, Hu4x from 40 days of drought, Ro4x and Ro8x from 50 days of drought). Ellipses depict one standard deviation of the data (*α* = 0.68)

No significant difference between drought treatments was found by AMOVA (df = 5, ΦST = 0.0027, *p* value = 0.4330). Similarly, no significant correlation was found between drought duration and level of unmethylation (*r* = 0.67,* p* value = 0.1451) or full methylation (*r* = − 0.64,* p* value = 0.1727). PCoA did not reveal separation of samples of any drought treatment but showed the increase in variation of methylation pattern with the increase of drought duration (Fig. 7a).

AMOVA revealed significant differentiation between tetraploid and octoploid samples (df = 1, ΦST = 0.1153, *p* = 0.0001). Octoploid samples exhibited a higher proportion of unmethylated sites compared to tetraploid samples (0.12% vs. 0.08%), while the proportion of fully methylated sites was lower in octoploids than in tetraploids (0.37% vs. 0.41%). PCoA revealed substantial, though incomplete, separation of samples by ploidy level (Fig. 7b). Genotypes Ru8x and Hu8x had the most distinct methylation patterns (Suppl. Fig. 1).

## Discussion

### Response to drought

The molecular response to drought in plants involves a complex network of pathways and gene expression changes, which interact with other stress-response mechanisms, creating a highly integrated system. Several key proteins have been previously identified in drought-stressed plants, including late embryogenesis abundant (LEA) proteins, desiccation stress proteins, abscisic acid-responsive proteins, enzymes involved in the synthesis of osmoprotectants (sugars, proteins, glycine betaine), cold-regulated proteins, detoxification enzymes, as well as numerous transcription factors (Tzvi et al. 2000).

We identified multiple upregulated genes previously reported to be associated with drought stress. Among them, two showed the highest upregulation on both the 20th and 30th days of drought across both ploidy levels (Fig. 4a). The first gene encodes α-aminoadipic semialdehyde synthase (AASS, upregulated 5.0- to 8.4-fold), an enzyme that plays a crucial role in lysine catabolism through the saccharopine pathway in plants. A gene of another protein from this pathway, saccharopine dehydrogenase (SCCPDH, IPR032095), was also upregulated for these groups of samples. The saccharopine pathway has been previously shown to be upregulated under osmotic stress, contributing to the production of the osmolytes proline and pipecolate, which aid in water-stress adaptation (Rodrigues et al. 2006; Arruda and Barreto 2020). Although AASS is not typically classified among the key water-stress proteins, its gene has been identified among a hub of upregulated genes under water stress in *Nicotiana tabacum* and *Hordeum vulgare* (Chen et al. 2019; Panahi and Golkari 2024).

The second most upregulated transcript (4.7- to 8.0-fold) encodes the LEA1 protein (IPR005513). LEA proteins are classified into eight subgroups of small, highly hydrophilic polypeptides: LEA1–LEA6, dehydrin, and seed maturation protein (SMP) (Hunault and Jaspard 2010). LEA proteins play a crucial role in protecting plant metabolism under abiotic stresses such as drought. Progressive drought and salinity stress induce LEA proteins in vegetative organs, where they contribute to stabilizing enzyme complexes and maintaining cell membrane integrity through antioxidant activity, metal ion binding, membrane and protein stabilization, hydration buffering, and interactions with DNA and RNA (Chourey et al. 2003; Goyal et al. 2005; Battaglia and Covarrubias 2013; Liang et al. 2019). In addition, two genes encoding other LEA proteins—dehydrin (IPR000167) and SMP (IPR007011)—were also found to be upregulated up to 4.4-fold and 3.0-fold, respectively.

A third most highly upregulated (up to 6.6-fold) gene under drought stress is encoding endo-1,4-β-xylanase (IPR001000), an enzyme involved in remodeling the plant cell wall by hydrolyzing xylan. Knockout studies in *Zea mays* have shown that loss of this gene results in thinner cell walls with reduced xylan and lignin content compared to wild-type plants. These structural deficiencies ultimately impaired water transport capacity and led to lower drought tolerance (Hu et al. 2020).

One more pathway was found to be involved in the drought response. Three different upregulated transcripts were assigned to the gene *HMGCR* encoding hydroxymethylglutaryl-CoA reductase (IPR009029, IPR009023, IPR002202), a key enzyme in the mevalonate pathway for isoprenoid biosynthesis. Isoprenoids play an important role in plant stress responses by providing protection against oxidative damage, thermal stress, and drought, while also contributing to stress signaling and gene regulation (Vickers et al. 2009; Zuo et al. 2019). Interestingly, *HMGCR* expression was significantly higher on the 20th day of drought compared to the 30th day, suggesting dynamic changes in response to increasing drought severity. This pattern aligns with the observations of Perreca et al. (2020), who reported that isoprene content in *Picea glauca* needles increased under mild drought stress but decreased under more severe drought conditions.

The pathways involved in drought and salt stress responses are known to substantially overlap in plants (Ha et al. 2014; Krasensky and Jonak 2012; Luo et al. 2015). Therefore, we expected gene expression patterns similar to those reported in previous studies on *P. australis* under salinity stress (Holmes et al. 2016; Jia et al. 2024). Surprisingly, only one gene, *dehydrin*, was consistently identified among the core upregulated genes. However, consistent with these studies and a study on *P. australis* grown under low water levels (Eller et al. 2014; Haldan et al. 2023), no oxidative stress-response genes, such as manganese superoxide dismutase (IPR019832/IPR019831/IPR036324) or glutathione peroxidase (IPR000889), were upregulated.

We identified additional upregulated genes that are potentially involved in drought stress. These genes encode non-symbiotic hemoglobin (NsHb, IPR000971), oil body-associated protein-like (OBAP-like, IPR010686), Bowman–Birk inhibitor (BBI, IPR000877), osmotin (IPR037176), heat shock-associated protein 32 (HSA32, IPR003830), β-fructofuranosidase (IPR021792), novel interactor of jasmonoyl (NINJA) or ABI five-binding protein (AFP) (both IPR032310), cold-regulated protein 413 (COR413, IPR008892), glycoside hydrolase (GH3, IPR002772), glyoxalase (GLO, IPR029068), and tyrosinase (Cu-binding domain, IPR002227).

### Photosynthesis under drought

Drought is known to suppress photosynthetic activity in plants by disrupting the balance between light capture and its utilization (Qiao et al. 2024). Previous studies have shown that *P. australis* tolerates mild water deficits by reducing leaf area while maintaining photosynthetic efficiency and RuBisCo activity through increased water-use efficiency (Pagter et al. 2005; Nada et al. 2015; Liu et al. 2018). However, more severe drought conditions impose biochemical limitations (Teng et al*.* 2022). In our previous mesocosm experiment, *P. australis* plants were grown with the water level 45 cm below the soil surface and exhibited no decrease in photosynthesis rate and expression of three key photosynthetic genes (*ribulose bisphosphate carboxylase, phosphoglycerate kinase,* and *phosphoribulokinase*) compared to those with water at the soil level (Haldan et al. 2023). This could suggest that the decrease in water availability was not severe enough to induce stress.

A progressive decline in the photosynthetic rate throughout the drought period was observed by Haldan et al. (2025; Fig. 3c) with the most pronounced decrease occurring within the first 20 days. Transcriptomic data showed that one of the genes, *PsbP*, was substantially downregulated, with a 22.7- and 34.9-fold change after 20 and 30 days of drought, respectively (Fig. 4b). This gene encodes the P subunit of the oxygen-evolving complex in Photosystem II (PSII), which oxidizes water to provide protons for Photosystem I (PSI). The PsbP subunit is thought to play a role in regulating the balance between water oxidation and plastoquinone reduction (Ifuku and Nagao 2021). Plastoquinone acts as an antioxidant, and its reduction status has been recognized as a key indicator of stress impact on photosynthesis (Havaux 2020; Sperdouli et al. 2021).

More photosynthetic genes were found to be downregulated, though less strongly. After 20 days of drought, *PsaO*, *PsaF*, and *carbonic anhydrase* were downregulated (2.3-, 2.3-, 4.3-, and 1.7-fold, respectively). On the 30th day of drought, *PsbO* (only for tetraploid samples) and *crr42* (protein chlororespiratory reduction) were downregulated 4.3- and 3.2-fold, respectively. Gene Ontology enrichment analysis revealed that on the 20th day of drought, the cellular component terms, PSI and PSI reaction center, were among the downregulated transcripts. On the 30th day of drought, terms related to the assembly of the NADH dehydrogenase complex (plastoquinone) and the mitochondrial respiratory chain complex I were identified.

### DNA methylation pattern under drought

It was previously shown that plants can exhibit increased variance in DNA methylation when exposed to ecological stressors (Ashapkin et al. 2020; Akhter et al. 2021). For example, genome-wide hypermethylation occurs in drought-stressed plants like *Morus alba* and *Populus trichocarpa*, with differentially methylated regions linked to stress-response genes (Sun et al. 2022). Moreover, it is shown that drought-tolerant varieties of these species maintain more stable methylomes, while drought-sensitive plants show greater methylation variability.

Despite our AMOVA results showing no significant differences between drought treatments, the PCoA revealed an increase in variance among samples as drought progressed. By the 40th and 50th days of drought, methylation patterns were the most scattered and the least scattered in control samples. The increased variation in DNA methylation may reflect stochastic epigenetic reprogramming in response to drought stress, leading to random and variable methylation changes as part of a plastic mechanism (Feinberg and Irizarry 2010). In addition, genotype-specific differences in drought response may also contribute to this variability, as previously observed in another clonally growing species, *Trifolium repens* (Rendina González et al. 2018).

Previous epigenetic studies on *P. australis* (but not using MSAP) showed that DNA methylation is implicated in response to salinity. Results indicated that DNA methylation levels tend to decrease with increased salinity (Petroff 2013; Song et al. 2022). We found no statistically significant correlation between drought duration and methylation level; however, similar to previous studies, the pattern was positive for unmethylation (*r* = 0.67) and negative for full methylation (*r* = − 0.64) levels.

The lack of significant difference in DNA methylation between drought levels can also be connected with the limitations of MSAP. The key limitation is that MSAP detects methylation status only at the site as a whole (CCGG) and does not provide single-base resolution (Hermawaty et al. 2024). The method is also sensitive to DNA quality, enzyme efficiency, and amplification conditions, which can affect reproducibility and accuracy. Originally, we included more samples in our study (leaf material before the treatment). However, DNA extracts were processed in two batches, three months apart. Exploratory data analysis via UPGMA dendrogram revealed a strong batch effect (samples formed two clusters), requiring the exclusion of one batch of samples. Future studies employing higher-resolution methods, such as bisulfite sequencing or epigenome-wide association studies (EWAS), could provide deeper insight into the relationship between drought stress and epigenetic regulation in *P. australis*.

### Difference between ploidy levels

Polyploid plants are often, though not always, shown to exhibit superior drought tolerance compared to their lower-ploid counterparts, attributed to enhanced abscisic acid signaling, improved photosynthetic and hormonal regulation, and increased antioxidant activity (Allario et al. 2013; Xue et al. 2015; Rao et al. 2020; Tossi et al. 2022; Correia et al. 2023). Their resilience under extreme stress conditions may also be linked to flexible reproductive strategies and the production of heavier, more viable seeds, particularly in grasses (Godfree et al. 2017; Stevens et al. 2020). However, our data on growth and photosynthesis, along with other common garden experiments on *P. australis* (Hansen et al. 2007; Achenbach et al. 2012), provide no evidence that octoploid plants outperform tetraploid plants under the same or stressful environmental conditions.

Previous transcriptomic analysis of three tetraploid and three octoploid *P. australis* genotypes identified differences in gene expression: tetraploid genotypes upregulated genes related to reproduction and defense against UV-B light and fungi, while octoploid genotypes upregulated genes associated with thermotolerance (Wang et al. 2021). However, no regional pairs were included in this study—all three octoploid plants originated from Australia, while tetraploid plants were collected from North America and Europe. Given the complex phylogenetic relationships within the species and the high variation in phenotypic plasticity and gene expression among genotypes and lineages (Achenbach et al. 2012; Eller et al. 2014; Zhang et al. 2020; Haldan et al. 2023), the observed differences may be driven by genotype-specific variation and local adaptation. To ensure robust comparisons of *P. australis* in future studies, a broader range of genotypes from diverse origins should be included.

Higher ploidy can significantly impact transcription and translation regulation due to increased genomic complexity. According to gene dosage theory, polyploidy is expected to elevate gene and transcript copy numbers, potentially leading to proportionally increased gene expression (Guo et al. 1996; Shi et al. 2015). However, some empirical studies report dosage compensation or non-additive gene expression, rather than a simple doubling of expression levels (Albertin et al. 2005; Wang et al. 2005). These findings suggest the existence of active regulatory mechanisms that buffer or normalize gene expression across ploidy levels (Chen and Ni 2006).

Polyploidy also results in the duplication of genes (homoeologs), creating opportunities for neo-functionalization (one gene copy acquires a new function) or sub-functionalization (ancestral functions are partitioned between gene copies). These processes contribute to the evolution of novel gene expression patterns, increasing regulatory and functional diversity that may enhance adaptive potential in polyploid organisms (Chen 2007; Jackson and Chen 2010).

In our study, upregulated genes identified in octoploid samples are mostly involved in translation regulation, but also in protein binding, and biosynthesis. These enriched biological processes can mitigate proteotoxic stress, ensure fidelity in protein synthesis, and support metabolic demands of larger cells. The most highly upregulated gene (7.2-fold) encodes the Mago Nashi protein, which plays a role in RNA metabolism, developmental signaling, and stress response in plants (He et al. 2007). To date, there is no direct evidence on ploidy-associated expression of this gene in the literature, but empirical studies are needed. Two genes found upregulated in tetraploid samples are involved in cell wall modification and transmembrane transport.

Tetraploid and octoploid samples responded differently to the drought. Between the 20th and 30th days of drought, tetraploid samples reduced the number of downregulated transcripts, while octoploid samples reduced the number of upregulated transcripts (Fig. 3a).

AMOVA and PCoA of MSAP data revealed differences in DNA methylation between tetraploid and octoploid samples, with octoploids exhibiting lower methylation levels. This difference is likely driven by genotype- or ploidy-related factors rather than drought response, as variation between treatments was smaller than that between genotypes (Suppl. Fig. 1). Another study using the MSAP method found that triploid *Citrullus vulgaris* and *Salvia miltiorrhiza* had 18–22% lower methylation levels than their diploid counterparts, whereas triploid *Populus* and *Pyrus* × *bretschneideri* exhibited 24–25% higher methylation levels than their diploid parents (Li et al. 2011). The absence of a linear relationship between ploidy and methylation levels suggests a complex, species-specific epigenetic regulation. In addition, evolutionary timing and hybridization type (allo- vs. autopolyploids) have a strong influence on methylation patterns (Yaakov and Kashkush 2011; Duan et al. 2023). Focusing on epigenetic modifications of stress-response genes, rather than genome-wide methylation, may provide more precise insights into the role of epigenetic changes in the stress adaptation of *P. australis* as a first step toward developing epigenetic control strategies for future hybridization and selection studies.

These findings have practical relevance for paludiculture, wetland restoration, and breeding for drought tolerance. The observed differences in gene expression and DNA methylation between ploidy levels highlight the diverse regulatory strategies that *P. australis* genotypes may use to cope with drought. Understanding these molecular responses can inform the selection of suitable genotypes for biomass production and wetland restoration under variable hydrological conditions.

## Conclusion

The molecular response to drought in *P. australis* involves a complex network of gene expression changes. We identified several key drought-response genes common to both ploidy levels, including those involved in the saccharopine pathway (*AASS* and *SCCPDH*), response to water deprivation (*dehydrin* and *LEA1*), cell wall remodeling (*endo-1,4-β-xylanase*), and mevalonate pathway (*HMGCR*). Drought suppressed photosynthesis, with a drastic downregulation of *PsbP*. Polyploidy influenced gene expression patterns, with octoploid samples upregulating genes involved in translation and metabolism, while tetraploids activated genes associated with cell wall modification and transmembrane transport. Ploidy levels displayed distinct gene expression dynamics throughout drought stress. Under non-stress conditions, a distinct gene expression pattern was observed, with different genes being differentially regulated. Notably, octoploid plants exhibited the strongest upregulation of the gene encoding the Mago Nashi protein. DNA methylation patterns showed increased variability under prolonged drought, although no significant correlation was found between methylation levels and drought duration. Pronounced differences in DNA methylation were found between tetraploid and octoploid plants, with octoploids exhibiting a lower methylation level. Given the complex phylogenetic relationships and genotype-specific responses of *P. australis*, future studies should incorporate a broader range of genotypes to improve comparisons. In addition, focusing on epigenetic modifications and other regulation mechanisms of stress-response genes could provide new insights into drought adaptation and potential epigenetic control strategies for hybridization, selection, and conservation studies.

## Supplementary Information

Below is the link to the electronic supplementary material.

Supplementary file1 (DOCX 88 KB)

Supplementary file2 (XLSX 3094 KB)

## Data Availability

Clean short and long reads, as well as assembled transcriptome, can be found on NCBI (PRJNA1195549). The details on data processing and analysis can be found on GitHub: https://github.com/kuprinak/Phragmites_drought_RNAseq.
