# Supplementary material for: How drought and ploidy level shape gene expression and DNA methylation in Phragmites australis
Source: Plant Cell Rep. 2025 Aug 12;44(9):197. doi: 10.1007/s00299-025-03585-9 (PMC12343755; doi:10.1007/s00299-025-03585-9)
Supplement: Supplementary file 1 — Supplementary file1 (DOCX 88 KB) [file 299_2025_3585_MOESM1_ESM.docx]

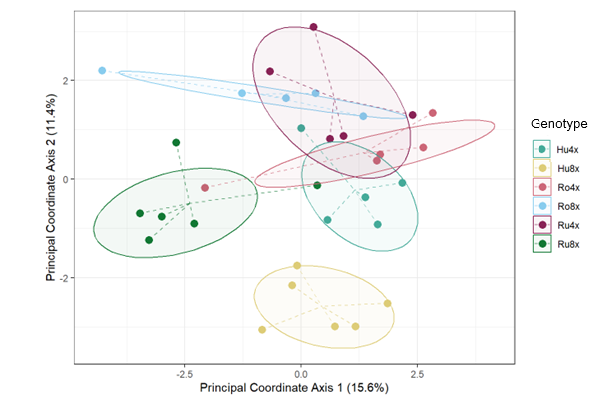


Supplementary Figure 1. Ordination diagram of PCoA calculated on 248 methylation-sensitive loci for 32 leaf samples of six genotypes of *Phragmites australis*. Ellipses depict one standard deviation of the data (α = 0.68).
